# Gleanings from Our Exchanges

**Published:** 1874-04-15

**Authors:** 


Dr. E. P. Hurd, of Newburyport, Mass., in his address before the N. H. Medical Society, states that he has found chloral hydrate especially useful in the insomnia of infants. One grain may be given to a restless infant every hour till sleep is induced. Gelseminum admirably fulfills many of the requirements of a hypnotic, for its action seems to be largely that of an exalter of sympathetic function, and it lessens cerebral congestion. Three drops of tincture of gelseminum, with three of laudanum, and ten grains of bromide of potassium, every two hours, has succeeded in breaking up insomnolence when other remedies have failed.—*Bost. Med. & Sur. Jour.*

---

New York has formed a "Mutual Cremation Society."

to use the perchloride of iron injection, or swabbing, without having first tried the ordinary means; and that no one will be deterred, when these have failed, from promptly employing it, and thus giving his patient a last chance, on account of anything against its use, which we, in our impartial position, have thought it our duty to publish.

---

DISEASE OF THE HEART—PREGNANCY—ABORTIONS.—A woman, aged forty, having been pregnant fourteen times, a cardiac affection became apparent in the course of the fifteenth, which, with the sixteenth and seventeenth, ended by abortion in the sixth month. She enjoyed good health during the first fourteen pregnancies, which resulted in six abortions, six children born alive at the full term, and one still-born; but when she became *enceinte* the fifteenth time, she was attacked with beatings of the heart and great dyspnœa, which increased in violence till miscarriage occurred, when they stopped. In the next two pregnancies, the palpitation of the heart and dyspnœa were more frequent: they ceased each time on abortion taking place, when the children were expelled dead. The palpitations are accompanied by a very acute pain, like " cuts with a knife," on the left side of the chest, sometimes on the right; the pain reaches as high as the left clavicle, and down to the lower ribs through the axillary region. No pain in neck, arm, nor abdomen. Walking against the wind, the smell of cooking or of tobacco, induces these attacks of pain. She has no gastric disturbance. Pressure at the third rib, and at the insertion of the sterno-mastoid on left side, causes pain. There is a rough murmur accompanying the first sound of the heart, with slight friction sounds at the base.

Although not yet noticed in midwifery works, accoucheurs are aware of the bad influence of pregnancy on diseases of the heart. By the mere fact of pregnancy, the quantity of blood in a woman is increased, causing a physiological hypertrophy of the left ventricle. The mother's heart and lungs, henceforth, act for two persons. If mitral insufficiency exist there will be engorgement of lungs and left auricle, causing catarrhal diseases and bronchitis with hemoptysis. This is followed by intermittent pulse and cardiac asthænia.

Such are the effects of pregnancy on mitral insufficiency.

Now, in the face of these facts, may we not ask whether heart disease does not affect pregnancy; and whether in certain cases, no doubt rare, abortions and premature labors are not induced? — *Le Progres Medical.—Obstet. Jour. of Great Britain and Ireland.*

---

THE LOCAL TREATMENT OF PULMONARY CAVITIES BY INJECTIONS THROUGH THE CHEST WALLS.—Prof. Wm. Pepper gives, in the *Medical Times* for March 14, an account of some experiments in the treatment of pulmonary cavities by injections through the chest walls, the cavities first being emptied by the aspirator. He employed the smallest canula (No. 1) which accompanies Dieulofog's aspirator. The needle was introduced to a depth of from one-half an inch to two inches, over the region where the physical signs gave evidence of the existence of a cavity. A few drops of thin, watery pus were withdrawn in one instance, and a little blood followed the puncture in one or two other cases. The only fluid which he had as yet tried as an injection, was a dilute Lugol's solution (min. iv. to f. drachm i.), of which from four to ten minims were injected.

The principal point demonstrated by these trials, was the entire harmlessness of the procedure. Further trial is necessary to demonstate its usefulness.

Prof. Masler, of Greifswald, Germany, has also been experimenting in the same direction. Brief references to some of his cases, reported in the *Berliner Klinische Wochenschrift*, have appeared in the *Medical Times and Gazette* (London), Feb. 14, 1874.

